# Maternal-fetal outcomes in patients with immune-mediated inflammatory diseases, with consideration of comorbidities: a retrospective cohort study in a large U.S. healthcare system

**DOI:** 10.1016/j.eclinm.2024.102435

**Published:** 2024-02-01

**Authors:** Yeon Mi Hwang, Qi Wei, Samantha N. Piekos, Bhargav Vemuri, Sevda Molani, Philip Mease, Leroy Hood, Jennifer Hadlock

**Affiliations:** a Institute for Systems Biology, Seattle, WA, USA; b University of Washington, Seattle, WA, USA; c Providence Health and Services and Affiliates, WA, USA

**Keywords:** Autoimmune disease, Pregnancy, Immunomodulatory medications, Immune mediated inflammatory disease, Multiple chronic conditions

## Abstract

**Background:**

Immune-mediated inflammatory diseases (IMIDs) are likely to complicate maternal health. However, literature on patients with IMIDs undergoing pregnancy is scarce and often overlooks the presence of comorbidities. We aimed to evaluate the impact of IMIDs on adverse pregnancy outcomes after assessing and addressing any discrepancies in the distribution of covariates associated with adverse pregnancy outcomes between patients with and without IMIDs.

**Methods:**

We conducted a retrospective cohort study using data from an integrated U.S. community healthcare system that provides care across Alaska, California, Montana, Oregon, New Mexico, Texas, and Washington. We used a database containing all structured data from electronic health record (EHRs) and analyzed the cohort of pregnant people who had live births from January 1, 2013, through December 31, 2022. We investigated 12 selected IMIDs: psoriasis, inflammatory bowel disease, rheumatoid arthritis, spondyloarthritis, multiple sclerosis, systemic lupus erythematosus, psoriatic arthritis, antiphospholipid syndrome, Sjögren's syndrome, vasculitides, sarcoidosis, and systemic sclerosis. We characterized patients with IMIDs prior to pregnancy (IMIDs group) based on pregnancy/maternal characteristics, comorbidities, and pre-pregnancy/prenatal immunomodulatory medications (IMMs) prescription patterns. We 1:1 propensity score matched the IMIDs cohort with people who had no IMID diagnoses prior to pregnancy (non-IMIDs cohort). Outcome measures were preterm birth (PTB), low birth weight (LBW), small for gestational age (SGA), and caesarean section.

**Findings:**

Our analytic cohort had 365,075 people, of which 5784 were in the IMIDs group and 359,291 were in the non-IMIDs group. The prevalence rate of pregnancy of at least 20 weeks duration in people with a previous IMID diagnosis has doubled in the past ten years. 17% of the IMIDs group had at least one prenatal IMM prescription. Depending on the type of IMM, 48%–70% of the patients taking IMMs before pregnancy continued them throughout pregnancy. Overall, patients with one or more of these 12 IMIDs had increased risk of PTB (Relative risk (RR) = 1.1 [1.0, 1.3]; p = 0.08), LBW (RR = 1.2 [1.0, 1.4]; p = 0.02), SGA (RR = 1.1 [1.0, 1.2]; p = 0.03), and caesarean section (RR = 1.1 [1.1, 1.2], p < 0.0001) compared to a matched cohort of people without IMIDs. When adjusted for comorbidities, patients with rheumatoid arthritis (PTB RR = 1.2, p = 0.5; LBW RR = 1.1, p = 0.6) and/or inflammatory bowel disease (PTB RR = 1.2, p = 0.3; LBW RR = 1.0, p = 0.8) did not have significantly increased risk for PTB and LBW.

**Interpretation:**

For patients who have been pregnant for 20 weeks or greater, the association between IMIDs and adverse pregnancy outcomes depends on both the nature of the IMID and the presence of comorbidities. Because this study was limited to pregnancies resulting in live births, results must be interpreted together with other studies on early pregnancy loss and stillbirth in patient with IMIDs.

**Funding:**

10.13039/100000002National Institutes of Health.

**Research in context**

**Evidence before this study**

Using the search terms ((“pregnancy”) AND (“immune-mediated inflammatory disease” OR “autoimmune disease” OR “inflammatory bowel disease” OR “rheumatoid arthritis” OR “multiple sclerosis” OR “psoriatic arthritis” OR “psoriasis” OR “systemic sclerosis” OR “spondyloarthritis” OR “systemic lupus erythematosus” OR “vasculitis” OR “sarcoidosis” OR “antiphospholipid syndrome” OR “Sjögren's syndrome”)), we searched PubMed up to May 15, 2023. No language restrictions were applied. We found several studies assessing adverse pregnancy outcomes in patients with immune-mediated inflammatory diseases (IMIDs) using electronic health records (EHRs) and insurance claims. However, most studies did not control adequately for comorbidities, used billing claims data instead of medical charts, and had a small sample size.

**Added value of this study**

This was a retrospective cohort study using EHRs of recent ten years from hospitals and clinics across seven western states in the USA and examined the association between IMIDs and the risk of adverse maternal-fetal health outcomes. To our knowledge, this is the first study that extensively controlled for multiple prevalent chronic comorbidities, including depression, as well as confounding variables for demographics, maternity characteristics and geographic socioeconomic factors, and present results for each IMID. This adds value to our analysis of the influence of IMIDs on maternal-fetal outcomes, as many previous studies insufficiently accounted for the contribution of confounding variables on outcomes. IMIDs pregnant patients doubled in recent ten years. They were more likely to be white, non-Hispanic or Latino, older, and have commercial insurance. They were two to three times more likely to have diabetes, asthma, obesity, urinary tract infection, sexually transmitted disease, and cardiovascular diseases. Most pregnant people with IMIDS who were prescribed immunomodulatory drugs before pregnancy continued their prescription throughout the pregnancy. Compared to matched control patients, IMIDs pregnant people had a slightly increased risk of preterm birth, small for gestational age, low birth weight, and caesarean section. The association between IMIDs and the increased risk of adverse pregnancy outcomes depended on the type of IMIDs and the presence of comorbidities. We found that, unlike prior studies, after 20 weeks of gestational age, neither rheumatoid arthritis nor inflammatory bowel disease showed increased risk for preterm birth, small for gestational age, or low birth weight when comorbidities were taken into consideration.

**Implications of all the available evidence**

The findings of this study reveal that the associations between IMIDs and adverse pregnancy outcomes are influenced by the specific type of IMIDs and the presence of comorbidities. Pregnant patients with IMIDs were two to three times more prone to experience other chronic comorbidities. However, after adjusting for these co-occuring health conditions, the risk of low birth weight and preterm birth in pregnant patients with inflammatory bowel disease and rheumatoid arthritis was significantly reduced. There is a need to take comorbidities into consideration for guidelines for patients with inflammatory bowel disease and rheumatoid arthritis and when designing future research to investigate maternal health in patients with IMIDs.

## Introduction

Immune-mediated inflammatory diseases (IMID) are a group of conditions with heterogeneous clinical presentation that share some common pathogenic immune pathways and affect multiple human body organ systems.^1^ IMIDs are generally characterized by organ damage and chronic inflammation, resulting in reduced quality of life, comorbidities, and premature death.^2^ Although each individual IMID has unique epidemiology and pathophysiology, its pathogenesis is primarily attributable to an imbalance in immune cellular activation and inflammatory cytokines.^1^ The underlying causes and mechanisms for the pathogenesis of many IMIDs remains ill-defined, but there have been significant therapeutic advances over the past two decades.^1^ We investigated a partial list of IMIDs in this study: psoriasis (PsO), inflammatory bowel disease (IBD), rheumatoid arthritis (RA), spondyloarthritis (SpA), multiple sclerosis (MS), systemic lupus erythematosus (SLE), psoriatic arthritis (PsA), antiphospholipid syndrome (APS), Sjögren's syndrome (SjS), vasculitides (Va), sarcoidosis (Sa), systemic sclerosis (SSc) (order based on number of IMIDs diagnosis in our study).

Although the degree of sexual bias varies widely across individual IMIDs, most IMIDs occur more frequently in females than males; 80% of patients with autoimmune diseases are female.^3^ RA, IBD, MS, SLE, APS, SjS and SSc occur 2–6^4^ (RA), 1.5^5^ (IBD), 2^6^ (MS), 7–10^7^ (SLE), 3.5^8^ (APS), 13^9^ (SjS), and 3–8^10^ (SSc) times more often in females than males. Given insufficient understanding of the pathology and mechanisms of IMIDs, the underlying reason for sexual dimorphism in IMIDs is still unknown.

It is particularly important to evaluate the relationship between pregnancy and IMIDs because IMIDs are often first diagnosed during reproductive age. Pregnancy can ameliorate or exacerbate disease activity, depending on the specific IMID.^11^ Both MS and RA can improve during pregnancy and flare after the delivery.^11^ SLE can induce unpredictable changes in disease activity during pregnancy and is one of the most significant risk factors for adverse pregnancy outcomes.^11^, ^12^, ^13^, ^14^ Less common autoimmune rheumatic diseases (e.g., PsA) were also shown to associate with worse outcomes including preterm birth (PTB), small for gestational age (SGA).^15^ Recent meta-analysis studies on RA and IBD reported elevated risk of adverse pregnancy outcomes including PTB, low birth weight (LBW), caesarean section or stillbirth.^16^^,^^17^ Pregnancy itself is a significant perturbation in the maternal immune system; the maternal immune system has to avoid rejecting a semi-allogeneic fetus while remaining immunocompetent.^18^ Because IMIDs can further complicate pregnancy and patient’s health, patients often voluntarily avoid pregnancy. Patients with IBD had significantly higher rate of voluntary childlessness, ranging from 14 to 18%, compared to 6.8% of the general population.^19^ However, recent years have shown improvements in pregnancy outcomes through substantial progress in diagnosis, and in preconception and prenatal care.^20^

Comorbid conditions are common in patients with IMIDs, including cardiovascular disease, metabolic and bone disorders and cognitive deficit.^1^ Also, patients with IMIDs have increased incidence of psychiatric disorders including depression, anxiety, and bipolar disorder, compared to geographically-, age-, and sex-matched controls.^21^ Despite the high cooccurrence of comorbidities among patients with IMIDs condition, the impact of comorbidities on the relation between IMIDs and pregnancy course is insufficiently examined.

The objective of this study is to retrospectively characterize patients who had one or more diagnoses of IMIDs prior to pregnancy regarding their demographics, pregnancy characteristics, comorbidities, and use of immunomodulatory drugs. We will evaluate the impact of IMIDs on adverse pregnancy outcomes after assessing and addressing any discrepancies in the distribution of covariates associated with adverse pregnancy outcomes between patients with and without IMIDs. We will also conduct disease-specific and sensitivity analyses to examine the contribution of individual IMIDs and comorbidities on the relationship between IMIDs and adverse pregnancy outcomes.

## Methods

### Study setting and participants

**Fig. 1:**
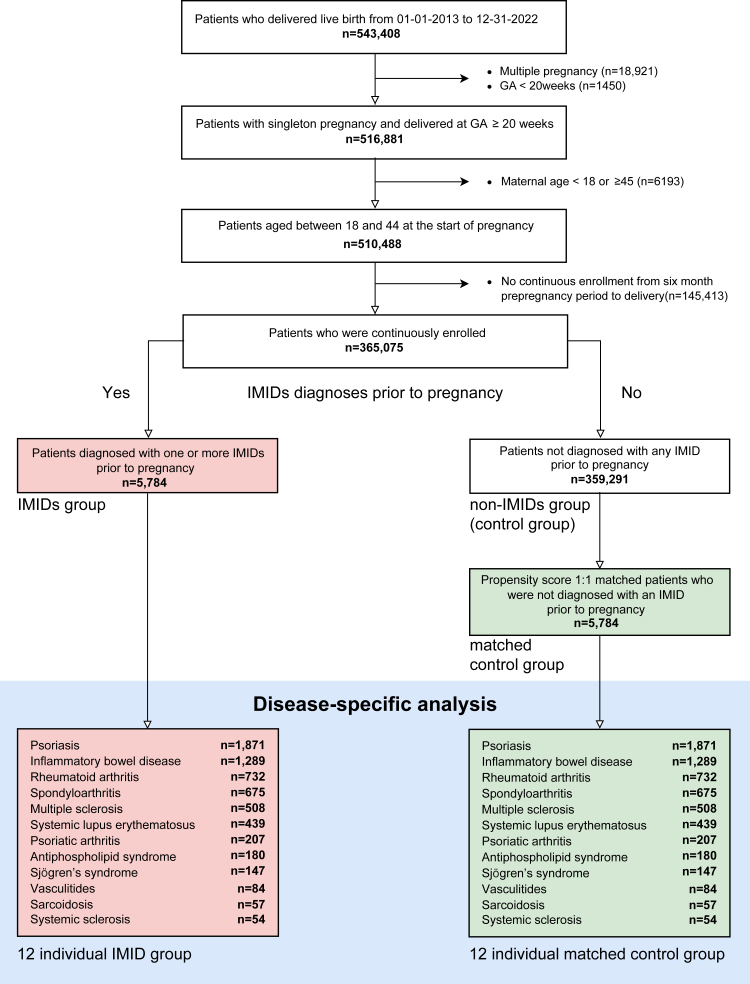
**Cohort selection flow chart.** IMIDs group was propensity score matched 1:1 on confounding variables to generate the matched non-IMIDs group. Individual IMID groups were propensity score matched 1:1 on pregnancy/maternal characteristics and comorbidities variables to generate corresponding matched control groups. GA, gestational age; IMIDs, immune-mediated inflammatory disease.

Providence St Joseph Health (PSJH) is an integrated U.S. community healthcare system that provides care in urban and rural settings across seven states: Alaska, California, Montana, Oregon, New Mexico, Texas, and Washington. We used a database containing all structured data from PSJH electronic health records (EHRs) and analyzed the cohort of pregnant people who had live births from January 1, 2013, through December 31, 2022 (n = 543,408). Medication prescribed and diseases diagnosed outside of PSJH are noted in records to the extent that patients report them when asked during encounters. Fig. 1 describes the cohort selection. We included pregnant people with age between 18 and 45 years (n = 510,488). Delivery records with missing information regarding the living status of born babies were excluded because these records are susceptible to higher rates of missing data and potential surveillance bias. Analysis was limited to records with live births to prevent potential underestimation of miscarriage and stillbirth cases. To reduce surveillance bias, we included people who had continuity of care at PSJH. Continuous enrollment was defined as at least one encounter 180 days prior to conception and one encounter on or after the delivery date. We excluded multifetal gestations and deliveries with gestational age (GA) of less than 20 weeks (n = 516,881). GA was limited to 20 weeks or greater because ascertainment bias is particularly high for EHR data earlier in pregnancy.

### Exposure and outcomes

Our exposure of interest was a clinical diagnosis of an existing IMID prior to pregnancy. The IMIDs group was people who had at least one type of IMIDs before pregnancy (n = 5784). The twelve IMIDs we investigated were PsO, IBD, RA, SpA, MS, SLE, PsA, APS, SjS, Va, Sa, and SSc. Systemized Nomenclature of Medicine (SNOMED) diagnoses of these IMIDs were clinically reviewed (Supplementary Table S1). People with no IMIDs diagnosis before pregnancy comprised the control group, the non-IMID group (Fig. 1).

Our outcomes of interest were adverse pregnancy outcomes: preterm birth (PTB; birth before 37 weeks of gestational age), low birthweight (LBW; birth of baby weighing less than 2,500 g), small for gestational age (SGA; birth of baby weighing less than 10 percentile of babies born in same gestational age), and caesarean section (Supplementary Table S2).

### Cohort characteristics

We collected information on maternal/pregnancy characteristics, comorbid conditions, and pre-pregnancy/prenatal immunomodulatory medications (IMMs) prescription pattern (Supplementary Table S2). The included maternal/pregnancy characteristics are parity, gravidity, history of preterm delivery, delivery year, fetal sex, maternal age, pregravid body mass index (BMI) category, self-reported smoking status, self-reported illegal drug use status, self-reported racial group, self-reported ethnic group, insurance, Centers for Disease Control and Prevention social vulnerability index (CDC-SVI), and rural-urban classification (Supplementary Table S2). CDC-SVI represents the percentile ranking of each census tract on 15 social factors. Social factor themes include socioeconomic status, household composition, race/ethnicity/language, and housing/transportation. CDC-SVI and rural-urban classification were collected based on the census tract patient resided in. Maternal and pregnancy characteristics were collected at the time of the prenatal visit. We included the following comorbidities: urinary tract infection, sexually transmitted disease, obesity, diabetes, asthma, depression, chronic kidney disease, chronic lung disease, pneumonia, and sepsis. Comorbidities that had been diagnosed by the start of pregnancy (last menstrual period; LMP) were observed. Missing values were imputed using median values, except for missing pregravid BMI, which was classified as normal pregravid BMI.

We assessed the pre-pregnancy and prenatal prescription patterns of IMMs. We defined prescription status based on prescription records within the period of interest. We limited our search to records with administration routes of oral, intramuscular, intravenous, subcutaneous, and rectal. The pre-pregnancy study period was 180 days from LMP to LMP. Prenatal medication exposure was further categorized based on their administration status in each trimester. IMMs we investigated were hydroxychloroquine, methotrexate, leflunomide and teriflunomide, 5-aminosalicyclic-acid derivative (5-ASA), azathioprine, mercaptopurine, mitoxantrone, mycophenolate, calcineurin inhibitors, tumor necrosis factor-alpha (TNF-α) inhibitor, fumarates, interferon, alkylating agent, hydroxyurea, dapsone, cladribine, interkeukin (IL)-1 inhibitors, IL-6 inhibitors, IL-12/23 inhibitors, IL-17 inhibitors, IL-12/23 inhibitors, IL-23 inhibitors, abatacept, anti-B lymphocyte stimulator (anti-BLyS), sphingosine 1-phosphate (S1P) receptor modulator, Janus kinase (JAK) inhibitor, phosphodiesterase-4 (PDE4) inhibitor, anti cluster of differentiation 20 (anti-CD20), anti-CD52, budesonide, and systemic glucocorticoids (Supplementary Table S3).

### Descriptive statistics

We described pregnancy/maternal characteristics, comorbidities, and pre-pregnancy/prenatal IMMs prescription patterns. We calculated the mean and standard deviation for continuous variables and the proportion of categories for binary and multi-class categorical variables (Supplementary Tables S4–S6). Differences in the distribution of variables between IMIDs and non-IMIDs groups were evaluated using the t-test, fisher exact test, and chi-square test for continuous, binary, and multi-class categorical variables (scipy v1.7.3). Multiple testing errors were corrected using the Benjamini–Hochberg method (statsmodel v.0.13.2) using the family-wise error rate α = 0.05.

### Propensity score matching

We generated a matched non-IMIDs group using the propensity score matching method. Propensity score was calculated across covariates using logistic regression (scikit-learn v1.0.2). Covariates include parity, gravidity, history of preterm delivery, delivery year, fetal sex, maternal age, pregravid BMI, race, ethnicity, insurance, pregravid BMI, smoking status, recreational drug use status, rural-urban classification, and comorbidities. People in the IMIDs group were 1:1 matched to the non-IMIDs group using the K-nearest neighbor algorithm in Euclidean metrics (k = 1), which identifies for each IMIDs person the most similar non-IMIDs person across several features in high-dimensional space (scikit-learn v1.0.2). We evaluated the balance across covariates after the matching using Cohen’s d value (Supplementary Figure S2, Supplementary Tables S7 and S8).

### Disease-specific analysis and sensitivity analysis

We replicated descriptive statistics and propensity score matching on individual IMIDs. Patients of individual IMID groups were 1:1 matched with non-IMIDs people to generate individual matched control groups (Supplementary Table S9). For sensitivity analysis, we conducted propensity score matching without comorbidities to examine the influence of comorbidities on the associations between IMIDs and adverse pregnancy outcomes (Fig. 1). Sensitivity analysis assessing the impact of comorbidities on the association was conducted on individual IMID groups (Supplementary Table S9). We additionally conducted a sensitivity analysis to confirm our assumption that patients with IMMs prescription during pregnancy had ongoing disease activities, affiliated with a higher chance of adverse pregnancy outcomes.^22^ We evaluated the risk of adverse pregnancy outcomes among patients with IMIDs compared to non-IMIDs group, looking at two subgroups: those with IMM prescriptions and those without.

### Ethics statement

All procedures were reviewed and approved by the Institutional Review Board at the PSJH through expedited review on 11-04-2020 (study number STUDY2020000196). Consent was waived because disclosure of protected health information for the study involved no more than minimal risk to the privacy of individuals.

### Role of the funding source

The funders had no role in study design, data collection and analysis, decision to publish, or preparation of the manuscript.

## Results

### Identification of IMIDs, individual IMID, and matched control cohorts

Our analytic cohort had 365,075 people, of which 5784 were in the IMIDs group and 359,291 were in the non-IMIDs group (Fig. 1, continuously enrolled singleton pregnant people). The IMIDs group’s most and least common IMID diagnoses were PsO and SSc, respectively (PsO n = 1871; IBD n = 1289; RA n = 732; SpA n = 675; MS n = 508; SLE n = 439; PsA n = 207; APS n = 180; SjS n = 147; Va n = 84; Sc n = 57; SSc n = 54). IMIDs people were 1:1 matched to non-IMID people to generate matched non-IMIDs groups (n = 5784), with standardized mean differences as shown in Supplementary Table S7.

### Characteristics of IMIDs and individual IMID cohorts

**Fig. 2:**
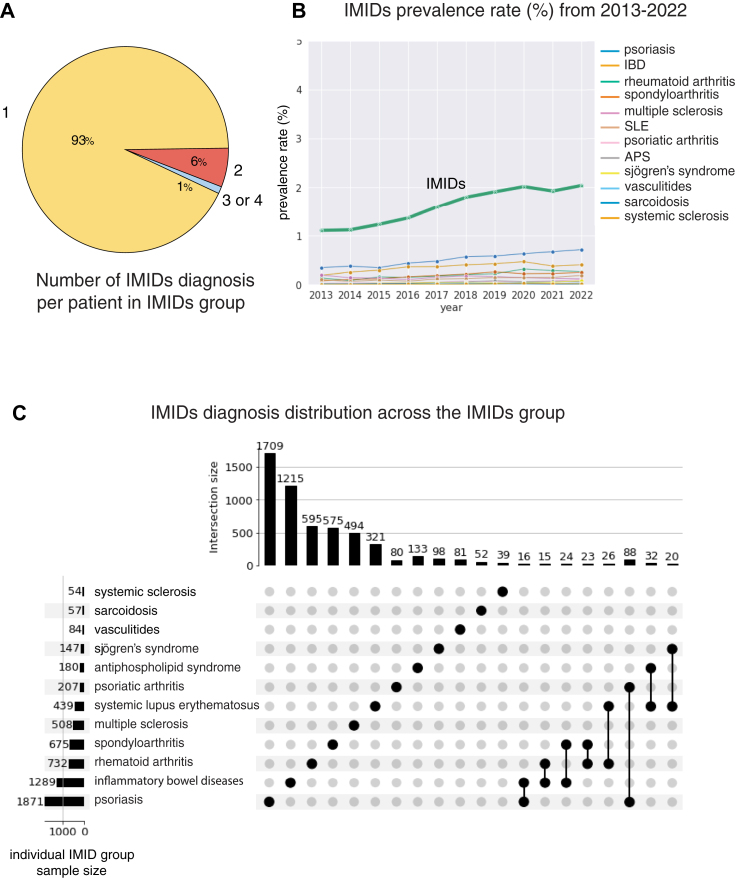
**Characteristics of the IMIDs group.** APS, antiphospholipid syndrome; IBD, inflammatory bowel disease; IMIDs, immune-mediated inflammatory disease; SLE, systemic lupus erythematosus. (A) Number of IMIDs diagnosis per patient. 93% of the IMIDs group had one IMID diagnosis. 7% of the IMIDs group had more than one IMID diagnosis. (B) IMIDs prevalence rate over time from 2013 to 2022. Prevalence rate was the proportion of patients with prior IMIDs delivered among patients delivered in the corresponding year. IMIDs prevalence rate gradually increased from 1% to 2% from 2013 to 2022, except in the year 2021. (C) IMIDs diagnosis distribution across the IMIDs group. The horizontal bars on the left indicates the sample size of the individual IMID group. The vertical bars on the tops are the number of patients who had the single IMID or combination of IMIDs shown in the corresponding column. Subsets size below 15 were not displayed. The most common and least common diagnosis was psoriasis (n = 1871) and systemic sclerosis (n = 54), respectively.

Fig. 1, Table 1 and Supplementary Table S4 describe non-IMIDs, IMIDs, and the subclassification of the IMIDs group into twelve distinct IMID groups. The prevalence rate of pregnancy with prior diagnosis of IMID increased from 1.1 to 2.0% in the recent ten years (Fig. 2). It escalated steadily from 2013 to 2020 and plateaued from 2020 to 2022. Compared to the non-IMIDs group, people in the IMID group (n = 5784) were more likely to identify as white (73.1% vs. 62.9%, p < 0.0001) or non-Hispanic or Latino (86.2% vs. 76.6%, p < 0.0001), be between the ages of 30–34 (54.8% vs. 43.1%, p < 0.0001), and have commercial insurance (55.2% vs. 46.3%, p < 0.0001). Regarding comorbidities, the IMIDs group had significantly more people with cardiovascular disease (35.0% vs. 13.9%, p < 0.0001), depression (22.1% vs. 9.1%, p < 0.0001), urinary tract infection (18.8% vs. 10.4%, p < 0.0001), asthma (12.0% vs. 5.2%, p < 0.0001), obesity (7.0% vs. 3.0%, p < 0.0001), sexually transmitted disease (3.5% vs. 1.7%, p < 0.0001), and diabetes (1.5% vs. 0.7%, p < 0.0001).

**Table 1:** Descriptive statistics of patients without and with IMIDs prior to pregnancy.

Variable	Patients without an IMIDs prior to pregnancy (n = 365,075)	Patients with an IMIDs prior to pregnancy (n = 5784)
**Pregnancy outcomes**		
Gestational age at birth (days)	275.0 (12.8)	274.0 (13.8)
Preterm birth	26,371 (7.7)	539 (9.3)
Low birth weight	18,365 (5.4)	393 (6.8)
Small for gestational age	41,316 (12.1)	778 (13.5)
Caesarean section	103,772 (30.2)	2012 (34.8)
**Maternal characteristics**		
**Maternal age**	31.0 (5.8)	32.7 (5.3)
**Age group**		
18–24	60,472 (17.6)	557 (9.6)
25–29	87,759 (25.6)	1225 (21.2)
30–34	112,308 (32.7)	2163 (37.4)
35–39	66,545 (19.4)	1478 (25.6)
40 or older	16,073 (4.7)	361 (6.2)
**Race group**		
American Indian or Alaska Native	4437 (1.3)	74 (1.3)
Asian	27,513 (8.0)	332 (5.7)
Black or African American	14,550 (4.2)	164 (2.8)
Native Hawaiian or other Pacific Islander	3953 (1.2)	48 (0.8)
White	215,895 (62.9)	4227 (73.1)
More than one race	17,127 (5.0)	290 (5.0)
Other	57,520 (16.8)	633 (10.9)
Missing	2162 (0.6)	16 (0.3)
**Ethnicity**		
Hispanic or Latino	72,607 (21.2)	665 (11.5)
Not Hispanic nor Latino	262,950 (76.6)	4986 (86.2)
Unknown/unreported	7536 (2.2)	133 (2.3)
**BMI category**		
Underweight	2823 (0.8)	62 (1.1)
Normal	49,346 (14.4)	985 (17.0)
Overweight	29,614 (8.6)	599 (10.4)
Obese	29,618 (8.6)	685 (11.8)
Missing	231,756 (67.5)	3453 (59.7)
**Insurance**		
Commercial	158,995 (46.3)	3191 (55.2)
Medicaid/Medicare	181,892 (53.0)	2575 (44.5)
Self pay	76 (0.0)	0 (0.0)
Missing	2194 (0.6)	18 (0.3)
**Smoker**	24,414 (7.1)	511 (8.8)
**Illegal drug user**	25,745 (7.5)	578 (10.0)
**Alcohol user**	60,516 (17.6)	1678 (29.0)
**Vulnerability index of socioeconomic status**	0.4 (0.2)	0.4 (0.2)
**Vulnerability index of housing composition**	0.4 (0.3)	0.4 (0.3)
**Vulnerability index of minority status and language**	0.6 (0.2)	0.6 (0.2)
**Vulnerability index of housing type and transportation**	0.6 (0.3)	0.6 (0.3)
**Rural/urban classification**		
Metropolitan	289,998 (84.5)	4952 (85.6)
Micropolitan	16,355 (4.8)	268 (4.6)
Small Town	5564 (1.6)	134 (2.3)
Rural	4378 (1.3)	84 (1.5)
Missing	26,862 (7.8)	346 (6.0)
**Pregnancy characteristics**		
**Gravidity**		
1	78,337 (22.8)	1240 (21.4)
2–4	189,217 (55.1)	3052 (52.8)
5≤	39,838 (11.6)	804 (13.9)
Missing	35,765 (10.4)	688 (11.9)
**Parity**		
0	42,250 (12.3)	717 (12.4)
1	121,735 (35.5)	2006 (34.7)
2–4	135,366 (39.4)	2254 (39.0)
5≤	8041 (2.3)	119 (2.1)
Missing	35,765 (10.4)	688 (11.9)
**History of preterm delivery**		
Yes	71,978 (21.0)	1232 (21.3)
No	235,414 (68.6)	3864 (66.8)
Missing	35,765 (10.4)	688 (11.9)
**Fetal sex**		
Female	156,624 (48.6)	2622 (48.4)
Male	165,401 (51.4)	2791 (51.5)
Unknown	48 (0.0)	2 (0.0)
Other	15 (0.0)	1 (0.0)
**Delivery year**		
2013	20,462 (6.0)	231 (4.0)
2014	30,362 (8.8)	346 (6.0)
2015	34,310 (10.0)	430 (7.4)
2016	35,708 (10.4)	495 (8.6)
2017	35,239 (10.3)	572 (9.9)
2018	34,887 (10.2)	640 (11.1)
2019	36,192 (10.5)	706 (12.2)
2020	34,680 (10.1)	715 (12.4)
2021	39,015 (11.4)	767 (13.3)
2022	42,302 (12.3)	882 (15.2)
**Comorbidities**		
Diabetes: type 1 or type 2	2510 (0.7)	88 (1.5)
Chronic kidney disease	218 (0.1)	18 (0.3)
Obesity	10,296 (3.0)	407 (7.0)
Chronic liver disease	661 (0.2)	30 (0.5)
Asthma	17,978 (5.2)	693 (12.0)
Depression	31,365 (9.1)	1276 (22.1)
Pneumonia	3072 (0.9)	119 (2.1)
Urinary tract infection	35,578 (10.4)	1086 (18.8)
Sexually transmitted disease	5712 (1.7)	203 (3.5)
Cardiovascular disease	47,737 (13.9)	2024 (35.0)
Sepsis	1491 (0.4)	81 (1.4)

BMI, body mass index; IMID, immune-mediated inflammatory disease.

Variables are defined in Supplementary Table S2. SNOMED codes of diagnoses are listed in Supplementary Table S1. Mean and standard deviation are calculated for continuous variables. Proportions of each category are calculated for categorical variables.

**Fig. 3:**
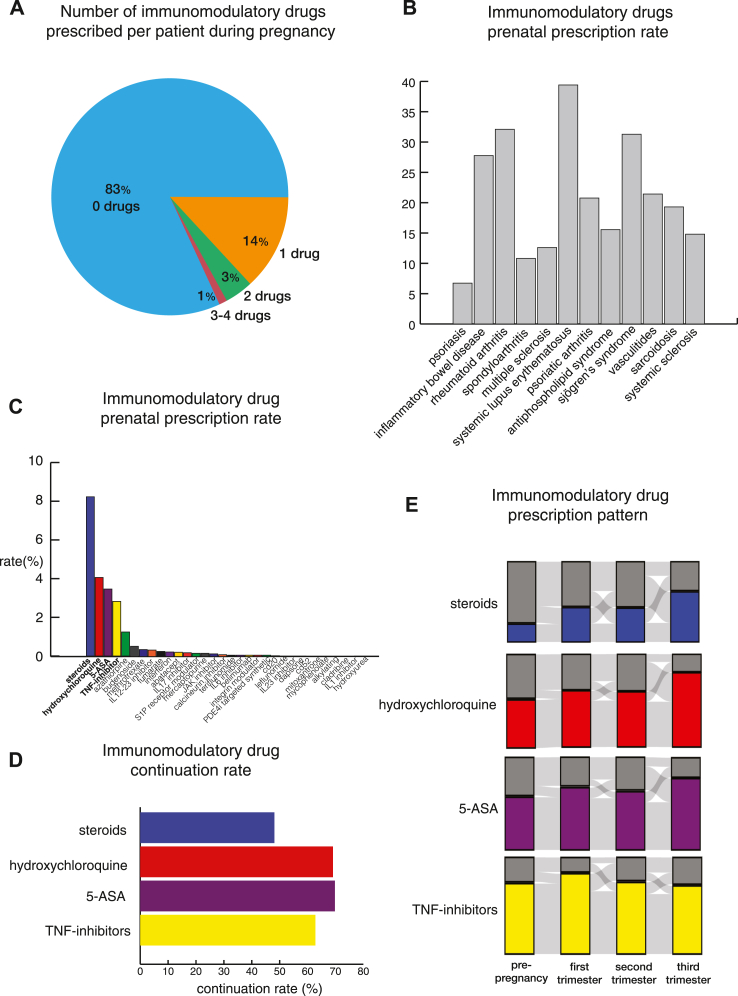
**IMMs prescription pattern of the IMIDs group.** 5-ASA, 5-aminosalicyclic-acid derivative; CD, cluster of differentiation; IL, interleukins; IMID, immune-mediated inflammatory disease; IMMs, immunomodulatory medications; JAK, Janus Kinase; LMP, last menstrual period; MS, multiple sclerosis; PsA, psoriatic arthritis; PsO, psoriasis; S1P, sphingosine-1-phosphate; Sc, sarcoidosis; SLE, systemic lupus erythematosus; SpA, spondyloarthritis; SjS, Sjögren’s syndrome; SSc, systemic sclerosis; TNF, tumor necrosis factor; Va, Vasculitides. (A) Number of IMMs prescribed per patient during pregnancy. 83% of the IMIDs group did not have any IMMs prescription. 17% had at least one IMMs prescription during pregnancy. (B) Prenatal IMMs prescription rate of individual IMID groups. The descending order of prenatal IMMs prescription rate of individual IMID groups were SLE (39.4%), RA (32.1%), SjS (31.3%), IBD (27.8%), Va (21.4%), PsA (20.8%), Sc (19.3%), APS (15.6%), SSc (14.8%), MS (12.6%), SpA (10.8%), and PsO (6.7%). (C) Prenatal IMMs prescription rate of the IMIDs group based on the type of IMMs. Glucocorticoids (steroids), hydroxychloroquine, 5-ASA, and TNF-α inhibitors were most commonly prescribed prenatally among the IMIDs group. Prenatal prescription rates were 8%, 5%, 4%, and 3%. Prenatal IMMs prescription rates of individual IMID groups based on the type of IMMs are displayed in Supplementary Figure S1. (D) IMMs continuation rate. Majority of patients, who were exposed to IMMs during the 180-day prepregnancy period, continued their prescription throughout the delivery. Continuation rates ranged from 48 to 70%. (E) IMMs prescription patterns among patients who prescribed corresponding IMMs at least once from LMP-180 days to delivery date. Pre, first, second, and third columns indicate 180 days prepregnancy period, first second and third trimester. Colored and gray portions, respectively indicate exposed and unexposed patients for corresponding time periods.

Fig. 2 describes the IMIDs cohort. 93% of people with IMIDs had one IMID diagnosis, and 7% of them had more than one IMID diagnosis. Fig. 3 characterizes the IMMs prescription pattern of the IMIDs cohort. 83% of IMIDs people had no prenatal IMMs prescription (Fig. 3A). Among the individual IMIDs we investigated, people with SLE and PsO had the highest and lowest IMMs prescription rates of 39.4% and 6.7% (Fig. 3B; SLE 39.4%, RA 32.1%, SjS 31.3%, IBD 27.8%, Va 21.4%, PsA 20.8%, Sc 19.3%, APS 15.6%, SSc 14.8%, MS 12.6%, SpA 10.8%, PsO 6.7%). Among patients with IMIDs, steroids, hydroxychloroquine, 5-ASA, and TNF inhibitors were the most commonly prescribed prenatal IMMs. Prescription rates were 8%, 5%, 4%, and 3% (Fig. 3C). The ranking of IMMs prescription rate varied depending on the type of disease (Supplementary Figure S1). For further investigation, we focused on these four medications as the rest had a prescription rate below 2%. The prescription rate of these IMMs increased in the first trimester when compared to that of pre-pregnancy (Fig. 3E). Most people prescribing these IMMs before the pregnancy continued taking them with a continuation rate ranging from 49% to 70% (Fig. 3D).

### Association between IMIDs and risk of adverse pregnancy outcomes

**Fig. 4:**
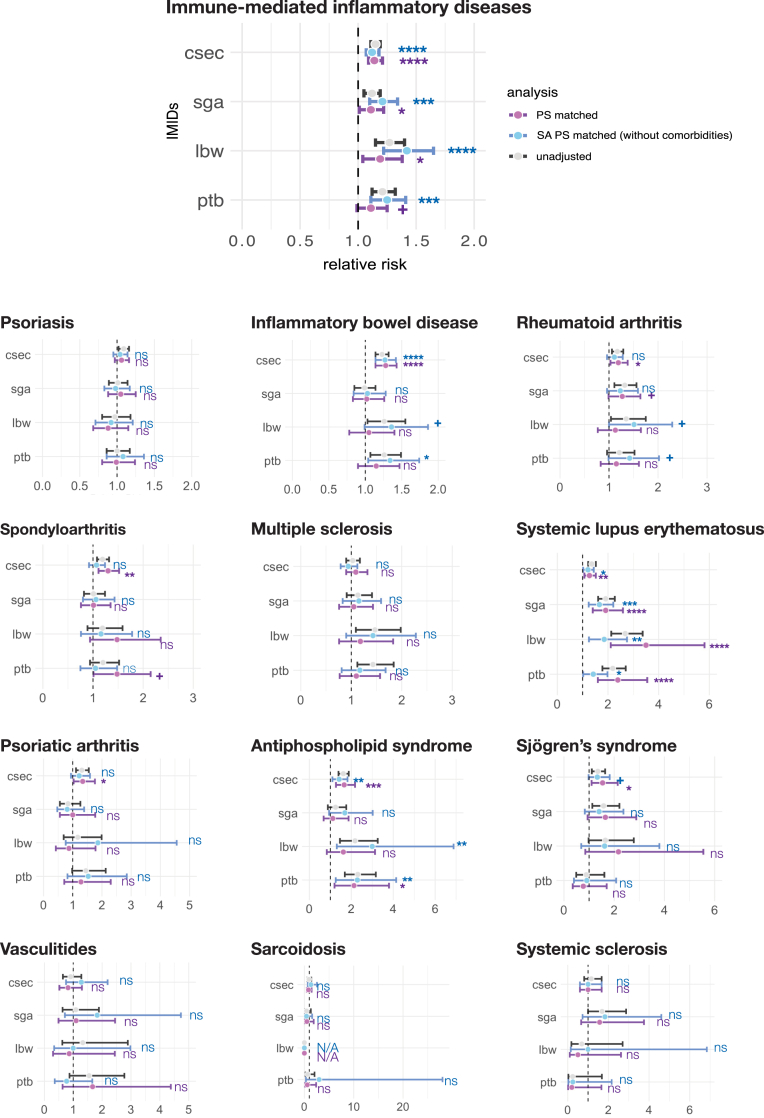
**Adverse pregnancy outcomes of the IMIDs group, propensity score matched non-IMIDs group, and sensitivity analysis propensity score matched non-IMIDs group.** APS, antiphospholipid syndrome; Csec, caesarean section; LBW, low birth weight; PTB, preterm birth; IBD, inflammatory bowel disease; IMID, immune-mediated inflammatory disease; IMMs, immunomodulatory medications; LMP, last menstrual period; MS, multiple sclerosis; PS, propensity score; PsO, psoriasis; PsA, psoriatic arthritis; RR, relative risk; Sc, sarcoidosis; SGA, small for gestational age; SLE, systemic lupus erythematosus; SpA, spondyloarthritis; SjS, Sjögren’s syndrome; SSc, systemic sclerosis; Va, vasculitides; Sensitivity analysis was performed to assess the influence of comorbidities on the association between IMIDs and risk of adverse pregnancy outcomes. The IMIDs group had slightly elevated risk of PTB (RR = 1.1 [1.0, 1.3]), LBW (RR = 1.2 [1.0, 1.4]), SGA (RR = 1.1 [1.0, 1.2]), and c-section (RR = 1.1 [1.1, 1.2]). Of 12 individual IMID, SpA, SLE, APS was associated with increased risk of PTB (SpA RR = 1.5 [1.0, 2.2]; SLE RR = 2.4 [1.6, 3.6]; APS RR = 2.1 [1.2, 3.8]). SLE was the only IMID correlated with enhanced risk of LBW (RR = 3.5 [2.1, 5.8]). RA and SLE patients were 1.3 ([1.0, 1.6]) and 1.9 ([1.4, 2.6]) times more likely to deliver SGA babies. IBD, RA, PsA, SpA, SLE, APS, and SjS patients had elevated likelihood of caesarean section delivery (IBD RR = 1.3 [1.1, 1.4], RA RR = 1.2 [1.0, 1.4], PsA RR = 1.3 [1.0, 1.8], SpA RR = 1.3 [1.1, 1.5], SLE RR = 1.3 [1.1, 1.5], APS RR = 1.7 [1.3, 2.2], SjS RR = 1.5 [1.1, 2.1]). When the comorbidities were not controlled, the IMIDs group’s risk of PTB and LBW increased by 0.2. In addition, the risk of PTB and LBW of IBD and RA patients increased and gained statistical significance (IBD: PTB RR = 1.3 [1.0, 1.7], LBW RR = 1.4 [1.0, 1.9]; RA: PTB RR = 1.4 [1.0, 2.0], LBW RR = 1.5 [1.0, 2.3]). The association between APS and the risk of LBW also elevated and became statistically significant (APS LBW RR = 3.0 [1.3, 6.9]). Statistical significance was reported as follows. p < 0.0001:∗∗∗∗, 0.0001 ≤ p < 0.001:∗∗∗, 0.001 ≤ p < 0.01:∗∗, 0.01 ≤ p < 0.05:∗, 0.05 ≤ p < 0.1:+, 0.1 < p:ns. RR, 95% confidence intervals, and p-value for results shown in Supplementary Figure S4 are in Supplementary Materials in Supplementary Table S9.

When we controlled for comorbidities, having an IMID was only weakly associated with the risk of PTB (RR = 1.1 [1.0, 1.3]), LBW (RR = 1.2 [1.0, 1.4]), SGA (RR = 1.1 [1.0, 1.2]), and caesarean section (RR = 1.1 [1.1, 1.2]) (Fig. 4, Supplementary Table S9). Of those 12 individual IMIDs, SpA, SLE, and APS were associated with increased risk of PTB (SpA RR = 1.5 [1.0, 2.2]; SLE RR = 2.4 [1.6, 3.6]; APS RR = 2.1 [1.2, 3.8]). SLE was the only IMID significantly correlated with enhanced risk of LBW (RR = 3.5 [2.1, 5.8]). People with RA and SLE were 1.3 ([1.0, 1.6]) and 1.9 ([1.4, 2.6]) times more likely to deliver babies with SGA condition. Patients with IBD, RA, PsA, SpA, SLE, APS, and SjS had a high likelihood chance of to deliver babies in caesarean section (IBD RR = 1.3 [1.1, 1.4], RA RR = 1.2 [1.0, 1.4], PsA RR = 1.3 [1.0, 1.8], SpA RR = 1.3 [1.1, 1.5], SLE RR = 1.3 [1.1, 1.5], APS RR = 1.7 [1.3, 2.2], SjS RR = 1.5 [1.1, 2.1]).

When we did not control comorbidities to assess the impact of comorbidities on associations between IMIDs and adverse pregnancy outcomes, having any IMIDs showed 0.2 higher RR of PTB and LBW (Supplementary Table S9). In addition, the risk of PTB and LBW of IBD and RA patients was higher and showed statistical significance (IBD: PTB RR = 1.3 [1.0, 1.7], LBW RR = 1.4 [1.0, 1.9]; RA: PTB RR = 1.4 [1.0, 2.0], LBW RR = 1.5 [1.0, 2.3]). Association between APS and the risk of LBW also elevated and became statistically significant (APS LBW RR = 3.0 [1.3, 6.9]). When compared with non-IMIDs group, patients with IMIDs and prenatal IMMs prescription had a higher RR across adverse pregnancy outcomes than patients with IMIDs and no prenatal IMMs prescription (Supplementary Table S10).

## Discussion

To our knowledge, this is the first study that extensively evaluated the influence of IMIDs and comorbidities on women undergoing pregnancy. Here, we retrospectively assessed people diagnosed with one or more IMIDs before pregnancy. People who had IMIDs before pregnancy doubled from 2013 to 2022. Of the 5784 pregnant people with IMIDs, only 17% were prescribed IMMs prenatally. Among people exposed to IMMs before the pregnancy, the majority, 48%–70%, continued their prescriptions until delivery. People who had an IMID diagnosis before pregnancy were more likely to have comorbidities than those who did not. Overall, patients with IMIDs had a similar but slightly elevated risk of adverse pregnancy outcomes after controlling for covariates when compared to controls. Patients with SLE had significantly elevated risk for all adverse pregnancy outcomes investigated. Out of the twelve selected IMIDs only three had an increased risk for PTB, one had an increased risk for LBW, two had an increased risk for SGA, and six had an increased risk for caesarean section. The risk of LBW and PTB of IMIDs was lower in analyses that controlled for comorbidities. We noted PTB and LBW risk on patients with RA or IBD are similar to patients without those conditions, unlike prior studies.

Prevalence rate of pregnancy with a history of IMIDs diagnosis doubled in the recent ten years. Several factors could explain this trend. First, maternal age increased. According to U.S. Census Bureau,^23^ fertility rate of 15–29 age range gradually dropped, whereas that of the 30–44 age range rose from 1990 to 2019. As IMIDs usually develop during the reproductive age and are untreatable, older people have a higher likelihood to have IMIDs at the time of pregnancy. Second, enhanced understanding about classification and diagnosis of IMIDs could partially explain the trend. This explanation was considered as a possible reason for increase in IMIDs prevalence rate in the general population.^24^ Last but not least, it could be attributable to actual increase in the IMIDs prevalence rate. Although the exact reason of prevalence rate is not identified, there is a consensus that IMIDs prevalence is gradually increasing.^25^

We found a strong association between specific IMIDs (SLE and APS) and adverse pregnancy outcomes. This finding is consistent with previous observations that SLE and APS are risk factors for adverse pregnancy outcomes.^26^ Gao et al., 2019^12^ built a deep learning model and predicted SLE as one of the most important features in predicting extremely preterm birth. A study conducted on the largest multicentric study to prospectively assess adverse pregnancy outcomes in SLE and/or APS pregnant patients (n = 385) reported nearly a 20% chance of adverse pregnancy outcomes, regardless of disease activity.^13^ Adverse pregnancy outcomes of that study were PTB, fetal/neonatal death, and fetal growth restriction. A meta-analysis of eleven observational case-control studies and thirteen cohort studies concluded that patients with SLE had twice the risk of delivering preterm when compared to controls.^14^

Surprisingly, most individual IMIDs (9: PTB, 11: LBW, 9: SGA, 6: caesarean section) were not correlated with adverse pregnancy outcomes. Given that autoimmune conditions are often considered a risk factor for high-risk pregnancy, this finding was somewhat unexpected compared to recent studies.^27^ A meta-analysis conducted on twelve studies and 3907 IBD patients showed a correlation between IBD and higher incidence of PTB, LBW, and caesarean section.^16^ Huang et al., 2023^17^ evaluated eighteen studies to assess maternal outcomes of pregnant patients with RA. That study reported elevated odds of PTB, SGA, LBW, and stillbirth among patients with RA. Our study differed from these studies because we extensively controlled for comorbidities, addressing both physical and mental conditions. IBD and RA correlation with PTB and LBW decreased and lost statistical significance when we included comorbidities in the covariates used for propensity score matching. This suggests that comorbidities significantly influence the relationship between IBD and RA, and PTB and LBW. Our result implies that, for several IMIDs, underlying comorbidities may be more significant risk factors than IMID itself. At the same time, the low rate of medication use in this cohort may suggest that the cohort had less severe cases of IMIDs, than populations studied in academic centers. To further understand IMIDs' relationship with adverse pregnancy outcomes, more research is needed to understand the trajectories of both IMIDs and comorbidities prior to pregnancy.

The observed co-occurrence of IMIDs within our study group was 7%. These results are likely lower than autoimmune co-occurrence for the overall population because the 12 IMIDs observed are a subset of all known IMIDs, and because pregnant women are significantly younger than the overall population. We observed a relatively low IMM prescription rate and a high continuation rate. The observation that 83% with an IMID prior to pregnancy had no prenatal IMMs prescriptions in the 180 days prior to pregnancy does not appear reflective of current recommendations for care.^11^^,^^28^ A meta-analysis conducted on 39 studies characterizing the use of TNF-α inhibitors among pregnant patients with IMIDs condition noted that 6% of them (1786/34,223) were exposed to TNF-α inhibitors during pregnancy.^29^ This finding somewhat accords with the prenatal TNF-α inhibitors prescription rate of our findings, 2.8%, considering that the majority of their samples were IBD patients (58% vs. 22%). The IMM prescription rate for patients with IBD was 1.6 times higher than for patients with IMIDs overall. The high continuation rate of IMMs corresponds with that prior study. Allen et al., 2022,^22^ a study on 338 patients with IMIDs, reported a 78% continuation rate of biologics, mostly TNF-α inhibitors. Our study found a similar rate of 70%.

The main strength of this study was the large sample size, use of real world longitudinal clinical observations from EHR data, and investigation of the relationship between several IMIDs and pregnancy. EHR contains comprehensive and longitudinal information including but not limited to medical history, diagnosis, prescription, location of residence, and socioeconomic factors. We could control for many factors known to be associated with adverse pregnancy outcomes and IMIDs factors, including maternal and pregnancy characteristics comorbid health conditions and socioeconomic environmental factors. To reduce surveillance bias, we limited our analyses to continuously enrolled patients. People transiently enrolled for pregnancy are likely to lack information on medical history, whereas people with IMIDs may be more likely to receive more continuous care from the health system. Therefore, people with IMIDs may have more detailed information on their health condition when compared to controls, leading to surveillance bias. In addition, we further addressed this potential bias by conducting propensity score matching when evaluating the outcomes. The propensity score matching method matches individual people with IMID to people with similar characteristics except for the exposure to IMIDs.

One major limitation of the study was that results were limited to live births with GA of 20 weeks or greater. Patients who experience early pregnancy loss or stillbirth may not always appear in their enrolled hospital’s system when their pregnancy ends, leading to significant risk of missing data, misclassification, and ascertainment bias. This could also have introduced selection bias toward fewer cases of adverse pregnancy outcomes in the cohort we investigated. Further research into early pregnancy loss before 20 weeks and stillbirth is essential but will require data beyond that available in EHR charts.

Another significant limitation of this study is that we did not evaluate the risks and benefits of IMMs exposure. The influence of IMMs on adverse pregnancy outcomes could not be properly addressed because information about IMID disease activity and severity was not available in structured EHR data. We assumed that people remaining on prescriptions were more likely to be experiencing ongoing disease activity, affiliated with a higher chance of adverse pregnancy outcome,^30^ which could induce confounding by indication. This assumption was further investigated in our sensitivity analysis. Patients with IMIDs who had IMMs prescriptions had higher RR across adverse pregnancy outcomes than patients with IMIDs who did not have IMMs prescriptions, but these associations did not reach statistical significance. The risks and benefits of IMMs exposure could be best addressed through prospective studies that collect data beyond that available in EHRs, or partially addressed by using severity information derived from free-text notes through natural language processing or manual abstraction. An additional limitation was the low IMM prescription rate. Although this reflects real world care in the population studied, results from this study may show higher risk than might be achieved with recommended care guidelines. Factors influencing provider and patient decisions may include barriers to access to healthcare or medication, differences in IMID activity or severity, hesitancy regarding taking medications during pregnancy, and other differences found between community and academic healthcare settings. Another limitation is that we considered the individual an independent entity, ignoring correlations among multiple pregnancies of a single person. As an alternative, we matched on parity and gravidity when generating matched control groups.

Because EHR data is not collected for research purposes, we expect it contains errors and omissions typically observed in patient charts, including potential underdiagnosis, overdiagnosis and misclassification of IMIDs. We believe most diagnostic data is sufficiently representative and matches expectation for this region, with the notable exception of obesity, a condition which has historically been underreported. Our results show 3% obesity in patients without IMIDs and 7% in patents with IMIDs, but in the states studied, prepregnancy obesity has been reported as up to 13.9%.^31^ We included information on pregravid BMI to offset underreporting of obesity but pregravid BMI also had a relatively high degree of missingness. However, we do not expect that this underreporting on either obesity or BMI was fundamentally different for patients with and without IMIDs. We therefore choose to still include the diagnosis and BMI data available for use in propensity score matching. Lastly, our resulting sample sizes for some IMID groups (SjS, Va, Sc, SSc) were too small to draw conclusions. This limitation was particularly important for vasculitides, which is a heterogeneous group of conditions with differing relationships to maternal-fetal health (Supplementary Table S1). As a result, we highlighted our findings on individual IMID groups with sufficient sample size and recommend future research with larger cohorts for IMIDs with lower prevalence.

Association between IMIDs and increased risk of adverse pregnancy outcome depended on the specific type of IMID and presence of comorbidities. After 20 weeks of gestational age, patients with RA or IBD had a similar likelihood to deliver preterm and low birthweight babies as patients without IMIDs. SLE or APS had strong associations with the adverse pregnancy outcomes we investigated. However, these findings need to be considered together with other research on early pregnancy loss and stillbirth, which were outside the scope of this study. Overall, there is a need to take comorbidities into consideration for guidelines for patients with RA and IBD, and when designing future research to investigate maternal health in patients with IMIDs.

## Contributors

Conceptualization: YH, QW, SP, BV, SM, JH. Resources: LH, JH. Data Curation: YH, QW, SP, BV. Methodology, Formal Analysis, Investigation, and Visualization: YH. Data Interpretation: YH, QW, SP, PM. Writing–original draft: YH. Writing–review and editing: YM, QW, SP, BV, SM, PM, LH. Supervision: LH, JH. All authors had final responsibility for the decision to submit for publication. YH, QW, BV and JH had full access to the data in the study and verified the data.

## Data sharing statement

All clinical logic has been shared. Results have been aggregated and reported within this paper to the extent possible while maintaining privacy from personal health information (PHI) as required by law. All data is archived within PSJH systems in a HIPAA-secure audited compute environment to facilitate verification of study conclusions.

## Declaration of interests

YH, SP, BV, QW and SM declare no conflict of interest. PM receives grant funding from Abbvie, Amgen, Bristol Myers Squibb, Eli Lilly, Galapagos, Gilead, Sun Pharma, UCB. PM consults for Abbvie, Acelyrin, Aclaris, Amgen, Boehringer Ingelheim, Bristol Myers Squibb, Eli Lilly, Galapagos, Gilead, GlaxoSmithKline, Inmagene, Moonlake Pharama, Novartis, Sun Pharma, Union Chimique Belge. PM is the Treasurer/Secretary of the Group for Research and Assessment of Psoriasis and Psoriatic Arthritis. JH has received research funding (paid to institute) from Pfizer, Novartis, Janssen, Bristol Myers Squibb and Gilead. LH is a scientific advisor for Sera Prognostics, a pregnancy diagnostics company. Sera Prognostics is not associated with this study or any of the findings.
